# Current Practice of Percutaneous Coronary Intervention in Patients With Coagulation Disorders

**DOI:** 10.7759/cureus.18284

**Published:** 2021-09-25

**Authors:** Michel El Khoury, Boutros Karam, Rabih Tabet, James C Lafferty, Stavros Thomas Snyder

**Affiliations:** 1 Internal Medicine, Staten Island University Hospital - Northwell Health, New York City, USA; 2 Cardiovascular Medicine, Staten Island University Hospital - Northwell Health, Staten Island, USA

**Keywords:** coagulation disorders, primary percutaneous intervention, : acute coronary syndrome, coronary artery angiogram, bleeding risk

## Abstract

Acute coronary artery disease represents the leading cause of death worldwide. Some studies have shown that coagulation disorders can play a protective role against ischemic heart disease, presumably due to hypocoagulable state and decrease thrombin formation. However, autopsy reports showed atherosclerotic lesions in some patients with hemophilia. Since the introduction of clotting factors and replacement therapies, the life expectancy of patients with coagulation disorders has increased significantly. As a result, the incidence of cardiovascular diseases became higher making their treatment more challenging. Door to balloon strategy applies in ST-elevation myocardial infarction (STEMI), and percutaneous coronary intervention should not be delayed. While in non-STEMI (NSTEMI) and unstable angina, a hematology consult is essential. Prophylactic coagulation factor replacement is crucial in these patients in order to avoid bleeding complications, but on the other hand, these factors were also associated with thrombotic complications. Historically, bare-metal stents were preferred over drug-eluting stents in view of the shorter duration of dual antiplatelets therapy (DAPT). Currently, some trials have demonstrated the safety of new-generation drug-eluting stents in patients with elevated bleeding risk, where DAPT use is limited to four weeks. The radial artery is the preferred access and was found to have less bleeding complications when compared to the femoral access. Anticoagulation with heparin is the safest in view of antidote availability and shorter half-life. Bivalirudin has also been used in some case reports, while GP2b3a inhibitors are usually avoided except in a high thrombus burden. Close peri procedural follow-up is important with patient education about symptoms of bleed. Carefully and individually tailored antithrombotic and factor replacement therapy is required to overcome these clinically challenging situations. Early screening for cardiovascular risk factors and considering early intervention and management might help to improve the general health status of this population and reduce morbidity.

## Introduction and background

We aimed to review the literature available for periprocedural and long-term strategies to both minimize the bleeding risk and ensure sufficient anticoagulation and anti-aggregation in patients with coagulation disorders undergoing coronary angiography with percutaneous coronary intervention (PCI). Among patients diagnosed with coagulopathic disorders, acute coronary syndrome treatment is challenging and exposure to antithrombotic agents and/or revascularization procedures may confer an enhanced risk of bleed. Currently, there are no clear guidelines for the treatment of ischemic heart disease in this population and the therapeutic approach is based on retrospective studies, expert opinion, and hospital-based guidelines. A systematic review was performed using PubMed. The following keywords were used in different combinations: “percutaneous coronary intervention,” PCI, hemophilia, von Willebrand, immune thrombocytopenic purpura, rare coagulation disorders. Relevant websites (i.e., clinicaltrials.gov; tctmd.com; cardiosource.com; theheart.org; escardio.org) and references of prior systematic reviews/meta-analyses were also screened for related studies. This article summarizes the available literature about the management of coagulation disorders including hemostatic therapeutic options and the specialized care needed for these patients. Lack of randomized clinical trials and the paucity of coagulopathic patients diagnosed with acute coronary syndrome limits trial design, data interpretation, and clinical decision-making. Therefore, comes the following question: Could lifestyle modification and preventive cardiology play a protective role in this high-risk population [1].

## Review

Hemophilia

Hemophilia A and B are sex-linked genetic deficiencies of coagulation factors VIII or IX, respectively. The clinical manifestations include spontaneous or provoked bleeding episodes. Since the introduction of clotting factors more than 50 years ago, the life expectancy of patients with hemophilia (PWH) has increased dramatically. An analysis of the Nationwide Inpatient Sample from 2007 found that the median age at death of hospitalized PWH in the United States was 68 years, compared to 72 years in hospitalized individuals without hemophilia [2]. As a result, the incidence of aging conditions like ischemic heart disease became higher. Currently, leading causes of death in PWH are similar to the general population including sepsis, pneumonia, and congestive heart failure, while traditional causes like intracranial bleed and HIV represents only a small proportion [3]. Among more than 3000 PWH from the US in the late 1990s, the prevalence of IHD ranged from 0.05% in those younger than 30 years to 15.2% in those more than 60 years [4]. That is why, preventive cardiology could play an important role in reversing modifiable risk factors like obesity, hyperlipidemia, and hypertension in PWH [5]. Autopsy reports on PWH with fatal myocardial infarction showed extensive atherosclerotic lesions, but only rarely fresh thrombi [6]. Sparse data are available for the management of acute coronary syndrome among PWH, but all experts agreed that coagulation factors must be replaced before any invasive procedure. Individuals with mild hemophilia usually require less factor concentrate replacement than do those with severe hemophilia and in some cases may not require any factor correction at all. This emphasizes an individualized approach, and the necessity for early hematology consult in order to navigate the fine balance between antithrombotic and hemostatic therapy required for these patients. According to Reilley et al. [7], patients with severe hemophilia presenting with ACS were most likely treated with medical treatment when compared to a control group in which PCI was commonly used. In this study, bare-metal stents (BMS) were used in the majority of patients. Rates of bleeding were higher in PWH treated with coronary artery bypass graft (CABG) and PCI, necessitating coagulation factors and blood products transfusion. But interestingly, there was no difference in mortality between the two groups. This might be secondary to selection bias, where clinicians usually preferred to treat mild cases of hemophilia with PCI while trying to avoid invasive approaches in moderate to severe cases. This would probably result in adverse cardiovascular outcomes. The door to balloon strategy also applies in PWH diagnosed with STEMI and intervention should not be delayed. Ideally, replacement therapy should run in parallel with ACS-specific treatment. Fibrinolysis can be used in the case early PCI (within 90 minutes) is not available, but clotting factors should be checked immediately aiming for a trough level of 50% and a peak of 80%. According to the World Federation of Hemophilia recommendations, PWH A who underwent major surgery (CABG) should be supplemented with factor VIII before the procedure to achieve a level of 80%-100% of factor VIII activity. However, there is no similar protocol for coagulation supplementation prior to PCI. Because hemophilia is not associated with platelets dysfunction, antiplatelets therapy use is crucial in PWH undergoing percutaneous transluminal coronary angioplasty and stent insertion in order to prevent stent thrombosis. Bovenzi et al. reported a case of acute stent thrombosis in a patient with hemophilia B after coronary stent implantation who was not treated with aspirin or clopidogrel [8]. Historically, BMS was preferred over drug-eluting stents (DES) in PWH because of the shorter duration of dual antiplatelets therapy (four weeks), but this would increase the risk of stent restenosis. Years ago, CABG was usually preferred over PCI when either option was feasible in PWH with stable coronary artery disease. A promising trial [9] favored the use of new second-generation DES over BMS in patients with high bleeding risk, where it showed a lower risk of MACE but interestingly no difference in all-cause cardiovascular mortality. This could limit the duration of dual antiplatelets therapy to 2-4 weeks, thus reducing the bleeding risk in a significant way. Unfortunately, this trial did not include any PWH. In the case of stable angina pectoris, a recommendation for aspirin use is less strong. If patients are using prophylaxis with clotting factor concentrates aiming for a trough of 5%, 80-100 mg of aspirin daily can be tried. In case of an increased bleeding frequency, aspirin should be stopped. If no clotting factor prophylaxis is given, we do not routinely prescribe aspirin. In urgent situations, such as acute STEMI, factor levels should be measured after the PCI, and subsequent replacement treatment made accordingly. Infusion of factor concentrates has been associated with precipitation of acute MI in some cases, that’s why a specialized hematologist should be consulted immediately in every single hemophilic patient diagnosed with ACS. Correction with clotting factor concentrates is mandatory post stent insertion. The minimum trough level should not fall below 15% to 30% while on dual antiplatelets. Ticagrelor and prasugrel were not studied in PWH and therefore their use is contraindicated. An international retrospective study was done by Fogarty et al. [10] with 54 hemophiliacs diagnosed with ACS, where 45 have type A and nine with type B. 38/54 (70%) had PCI while others were treated with CABG and medical treatment. 33/38 had stent insertion with 31 with BMS and two with DES, but only 28/33 were given DAPT for a median duration of one month. Coagulation factor supplementation was given on 21/28. Results were unpredictable, and it showed that one patient with mild hemophilia A who had a STEMI did not receive any secondary prophylaxis while on antiplatelet therapy, but he had experienced excessive gastrointestinal bleeding requiring discontinuation of ASA. An additional patient with severe hemophilia A and NSTEMI who was managed medically required an increase in FVIII replacement after two months of single-agent ASA, from 35 IU/kg twice a week to 25 IU/kg three times a week, due to nosebleeds and excessive bruising. Therefore, an individualized approach to PWH is mandatory. We have little data about the safety of Glycoprotein 2b/3a inhibitors use in PWH. Their use is limited to selected high thrombus burden situations [11] and not for routine use. They should never be given before factor replacement is performed. In case needed, a bolus of abciximab 0.25 mg/kg should be given, followed by 0.125 mcg/kg/min for 12 h with a maximum of 10 mcg/min. If tirofiban is used, a bolus of 10 mcg/kg should be given followed by 0.15 mcg/kg/min for 12 h. Heparin has successfully been used in PWH during coronary stenting [12]. It is preferred over low molecular weight heparin because of its shorter half-life, lower bleeding risk, and antidote availability. It should not be given before complete clotting factor correction is achieved. The dose is usually 70-100 IU/kg before PCI. Bivalirudin, a selective factor II antagonist, inhibits factor II reversibly and has a short half-life of 1.5 h. Currently, several case studies have shown that bivalirudin can be used safely with no complications during PCI in PWH [13]. A consensus about the management of ACS in PWH and the suggested therapeutic modalities concerning factor replacement has been adopted among physicians according to the ADVANCE working group recommendations [14]. The divergence of views regarding specific factor levels reflects the lack of absolute knowledge that would ideally be addressed in clinical studies designed to guide therapy in this area. Until such studies are undertaken, practice will continue to be based on experience rather than evidence. Two centers have published their experience-based algorithms for managing PWH who present with ACS [15,16]. They recommend aiming at a peak level of 0.8 U/L before PCI, and until 48 h after PCI. Higher levels should be avoided to prevent occlusive thrombi. This can be achieved by a slow bolus infusion of 40 U/kg FVIII or 80 U/kg FIX in 30 min, with an FVIII or FIX recovery assay 15 min after bolus infusion. A trough level of 0.5 U/L is pursued 48 h post PCI using slow bolus infusions of 20 U/kg FVIII or FIX every 12 h. The duration of clotting factor correction is dependent on the choice of the stent and the need for dual antiplatelet therapy with clopidogrel and aspirin. After the initial clotting factor infusion, as described above, we recommend clotting factor substitution aiming at trough levels of 0.3 U as long as dual antiplatelet therapy is given. Clotting factor measurements are required to optimize dosing. Afterward, the regular factor replacement schedule of the patient can be continued to keep a trough level of more than 5% while on aspirin. Concerning arterial access sites, radial access is preferred as it allows easier hemostatic compression because up to 70% of all major bleeding complications including access site hematomas and retroperitoneal bleed were reported in the femoral approach [17]. In fact, many publications documented catheterization via the femoral route in PWH and ITP patients with negligible complications [18]. In case femoral access is used, it is preferable to deploy a Suture-based closure device or collagen plug-based device [19], in order to reduce local bleeding complications. There was no RCT comparing between closure devices and manual compression in this population. The available evidence is limited to specific patient populations, often studied in a nonrandomized fashion, without methodological follow-up and standardized clinical outcomes. Successful deployment of these devices is highly dependent on the operator experience. Their use should be limited to catheterization with large French sheaths because placement failure was associated with vascular complications [20]. These complications were found more frequently with suture-based devices compared to collagen plug-based ones. In conclusion, given the available data from some case reports and some hospital-based guidelines, a similar approach to the general population must be performed in PWH presenting with ACS. We should consider prophylactic coagulation factor replacement in order to avoid bleeding complications. At all times, consulting a hematologist to help in the management is important. The radial artery is usually the preferred access site. Heparin is preferred over low molecular weight heparin and bivalirudin, in view of shorter half-life and antidote availability. Peak factor level must reach 80% during PCI and trough levels > 50% 48 hrs post PCI. Aspirin can be considered in stable ischemic heart disease considering a through factor level > 5%. DAPT is used after stent insertion and duration depends on the type of stent being used, with one month for BMS and six months at least for DES. Clopidogrel was the only P2y12 inhibitor studied in PWH. Trough level should be maintained > 30% during DAPT use.

Immune thrombocytopenic purpura

Moving next to patients with ITP, who were also found to have an increased risk of arterial and venous thrombosis. This was secondary to the presence of larger and more adhesive platelets, the release of thrombotic platelets microparticles, and antibody-mediated attack on endothelial cells caused by cross-reactivity. Due to the higher risk of bleeding complications observed in this population, rates of PCI were lower in patients with NSTEMI when compared to the general population, while rates of PCI were the same in patients with STEMI. This finding can be explained by the tendency for clinicians to lean toward conservative management in non-emergent conditions. Similar to hemophilia patients, BMS is preferred during PCI in view of a shorter period of DAPT, but two new randomized trials [9,21] have demonstrated the safety and superiority of second-generation DES when compared to BMS in patients with thrombocytopenia. In general, individual case reports have shown that aspirin and clopidogrel are well tolerated even chronically when the platelet count is above 30,000 [22]. According to Stouffer et al., only one patient developed petechiae and nasal bleed that required clopidogrel to be stopped [23]. A pooled analysis of three large Japanese studies by Ito et al. [24], showed that thrombocytopenia was associated with an increase of major bleeding events and all-cause mortality during the entire three-year follow-up period. On the other hand, a single-center retrospective study at Mayo Clinic showed no significant difference in inpatient bleeding and inpatient deaths after PCI in patients with thrombocytopenia and a matched control group. A review of literature done between 2007 and 2014 by Torbey et al. [25] showed no difference in mortality between the general population and ITP patients performing PCI, but there was a higher incidence of bleed especially in the ITP group admitted with STEMI. Patients who got transfused or who were treated with IVIg have more cardiovascular complications including thrombosis. Pre-treatment with steroids, IVIg, and platelets transfusion was required in 52%, 27%, and 13%, respectively. The mean platelet count at which PCI was performed was 145 ± 87,000. Major and minor bleeding occurred in 12% and 5% of cases, respectively. Heparin was used in 87% of cases (in six cases the anticoagulant was not mentioned). Femoral access was used in 80% of cases and hemostasis was achieved in 76% of cases by manual compression. Patients in this case series were discharged on single or dual antiplatelets in 83% of cases. According to Russo et al. [26], platelet count >50,000 was considered safe for invasive interventions including PCI and CABG in ITP patients. Individuals presenting with platelets < 30,000 or signs of active bleed should be pretreated with steroids and or IVIg while postponing the intervention if possible. Heparin is used in most cases with a good safety margin. Fondaparinux can be used as an alternative since it has no effect on platelet function. Bivalirudin is also a good alternative that is generally used in STEMI where the patient has low platelets and heparin cannot be given. Bivalirudin has also been used for a long time in heparin-induced thrombocytopenia. There is no clear data regarding the use of glycoprotein IIBIIIA in ITP despite two case reports that did not find any major bleeding events. Similar to hemophilia, radial access is the preferred site in view of easier hemostatic compression.

Von Willebrand

Von Willebrand disease is the most frequent congenital bleeding disorder in which there is either quantitative (type 1) or qualitative (type 2) defect in the von Willebrand factor (VWF). A study performed by Syed et al. [27] at Henry Ford Hospital between 1985 and 2010, found that six out of 198 patients with Willebrand disease were diagnosed with coronary artery disease, three with the acute coronary syndrome, and three with stable angina. The mean age at the time of diagnosis was 65.5 ± 10.6 years; 4/6 were females. Hypertension was seen in all of them, hyperlipidemia in three patients and four were smokers. VWF/FVIII replacement was given both before and after the procedure. The given dose of Humate-P was 50 U/kg body weight one hour before the procedure and subsequently the same dose 12 h after the procedure for the first 24-48 h. One female patient had a DES and she got DAPT for 12 months without any major bleed except for minor bruises at six months. Two patients got BMS in which clopidogrel was stopped after four weeks and two others underwent CABG for stenosis in the left main artery and proximal LAD, respectively. No major bleeding events were encountered in all these patients. This can be secondary to an increase in VWF levels with ACS, as a result of catecholamine surge, which may play a role in better tolerance of antiplatelet and antithrombotic medications [28]. In all patients, the femoral artery was the choice of access. In one reported case, a small groin hematoma was noted despite factor VWF/FVIII replacement [29]. Interestingly, occlusion of the LAD was observed in all six patients. Although coronary artery disease is rare in this population, when it does, it becomes a serious condition involving the main blood supply of the heart. Until now, no explanation exists for this observation. All of these patients have had type 1 von Willebrand disease that was corrected by factor VIII/VWF complex or cryoprecipitate infusion. Desmopressin is not preferred in view of the associated risk of thrombosis and hypertension. Similar to hemophilia and ITP, Tpa is in general contraindicated. Heparin or bivalirudin can be used and GP IIb/IIIa inhibitors use should be minimized. The same rule applies for patients with STEMI, where immediate revascularization is required with an aim to keep VWF activity level > 30%, while in patients with NSTEMI or unstable angina, a hematology consult is advised before proceeding with cardiac catheterization. Close peri procedural follow-up is required with patient education about symptoms of bleed. The femoral approach can be used if VWF > 30%.

Rare bleeding disorders

Other rare coagulation factor defects constitute 10% of all congenital bleeding disorders. Coagulation factor deficiencies are not protective against ACS. FXI was the most common coagulation factor deficiency described in the literature given its predominance in Ashkenazi Jews (1/1,000) but this does not mean that its deficiency is associated with coronary disease. According to a meta-analysis done by Girolami et al. [30], 53 patients with coagulation factor deficiency were diagnosed with ACS. Fibrinogen deficiency was diagnosed in four patients, FV deficiency in two patients, FVII defect in two patients; FXI defect in 36 patients; FXIII in one patient. Eight patients were diagnosed with rare platelets disorders: one with Glanzman thrombasthenia, three with Bernard Soulier syndrome, and four with MYH9 defects. The mean age was 60. Seven patients only were females. Hypertension was the most common risk factor (34%), followed by diabetes and dyslipidemia (26%) than smoking (19%). Six patients have no risk factors for CAD, and interestingly no ACS was described in FII or FX deficiency. The recent development and apparent success of the new oral anticoagulant with anti-FIIa or anti-FXa activity could be in agreement with the latter interpretation. However, this needs to be proven in future studies. Myocardial infarction secondary to factor replacement therapy can be fatal, and it was found in five patients. Four out of them died, three of them were treated with FXI concentrate. Genetic polymorphisms with specific mutations (e.g., Arg304Gln, Cys22Gly) were proven to play a role in the predisposition to thrombosis following factor concentrate infusion making the therapeutic approach more complicated [31]. Some studies have shown a protective role of FXI deficiency in ischemic stroke but not in myocardial infarction [32]. This indicates that elevated baseline PTT is not protective against coronary disease and the use of anticoagulation is necessary with stent insertion and it should be given under close monitoring of activated clotting time. On the other hand, Mungee et al. [33] demonstrated a favorable outcome in a patient who underwent percutaneous old balloon angioplasty where aspirin was the only medication used. Similar to other coagulopathy, fibrinolysis is not recommended, nor the use of Glycoprotein 2b/3a inhibitors. In unstable angina and NSTEMI, a hematology consult is recommended with prudent use of factor concentrates and FFP. Radial access should be tried first. A femoral access site was also used in many cases without major bleeding events and hemostasis was achieved by using a closure device. The real challenge is to maintain a fine balance between the risk of bleeding and thrombosis. Carefully and individually tailored antithrombotic and factor replacement therapy is required to overcome these clinically challenging situations.

Limitations

One of the most relevant limitations of this review is a search bias where a faulty search can miss some important studies. Small differences in search strategies can produce large differences in the set of studies found. In this review, we used the search engine PubMed with a list of keywords being used trying to make this list as complete as possible. Another important limitation is selection bias, where some case reports and meta-analyses were selected over others. This may be secondary to the paucity of published articles. In addition to that, lack of generalizability and the danger of overinterpretation is another major limitation of this review.

## Conclusions

In conclusion, sparse data are available for the management of acute coronary syndrome among patients with coagulation disorders. Therefore, assessment for modifiable cardiovascular and metabolic risk factors, beginning from early childhood, is crucial for this specific patient population. Studies have shown that obesity, hypertension, and metabolic syndrome are frequent especially among hemophiliac patients. This could be secondary to the negative effect of arthropathy on physical activity. Thus, early prevention and management of overweight, obesity, and their sequelae must be addressed in clinical practice in order to maximize the overall health of these individuals. There are no specific evidence-based guidelines that address the management of CVD risk factors in coagulopathic patients. It is not clear whether it is a true increase in cardiovascular risk or it is a result of better recognition of cardiovascular risk in the aging coagulopathic population. Larger multicenter prospective studies need to be conducted in order to detect the impact of early screening intervention on cardiovascular mortality in this population. Among many robust and well-established methods for early detection of vascular damage, measurements of carotid intima-media thickness and flow-mediated dilatation have been reported in hemophiliacs. Until then, collaborative efforts between primary care providers, cardiologists, and hemophilia center specialists are essential in managing this challenging group of patients.
